# Supplementary material for: Universal features of amorphous plasticity
Source: Nat Commun. 2017 Jul 3;8:15928. doi: 10.1038/ncomms15928 (PMC5500855; doi:10.1038/ncomms15928)
Supplement: Supplementary Information [file ncomms15928-s1.pdf]

Title of file for HTML: Supplementary Information
Description: Supplementary Figures and Supplementary Tables

Title of file for HTML: Peer Review File
Description:

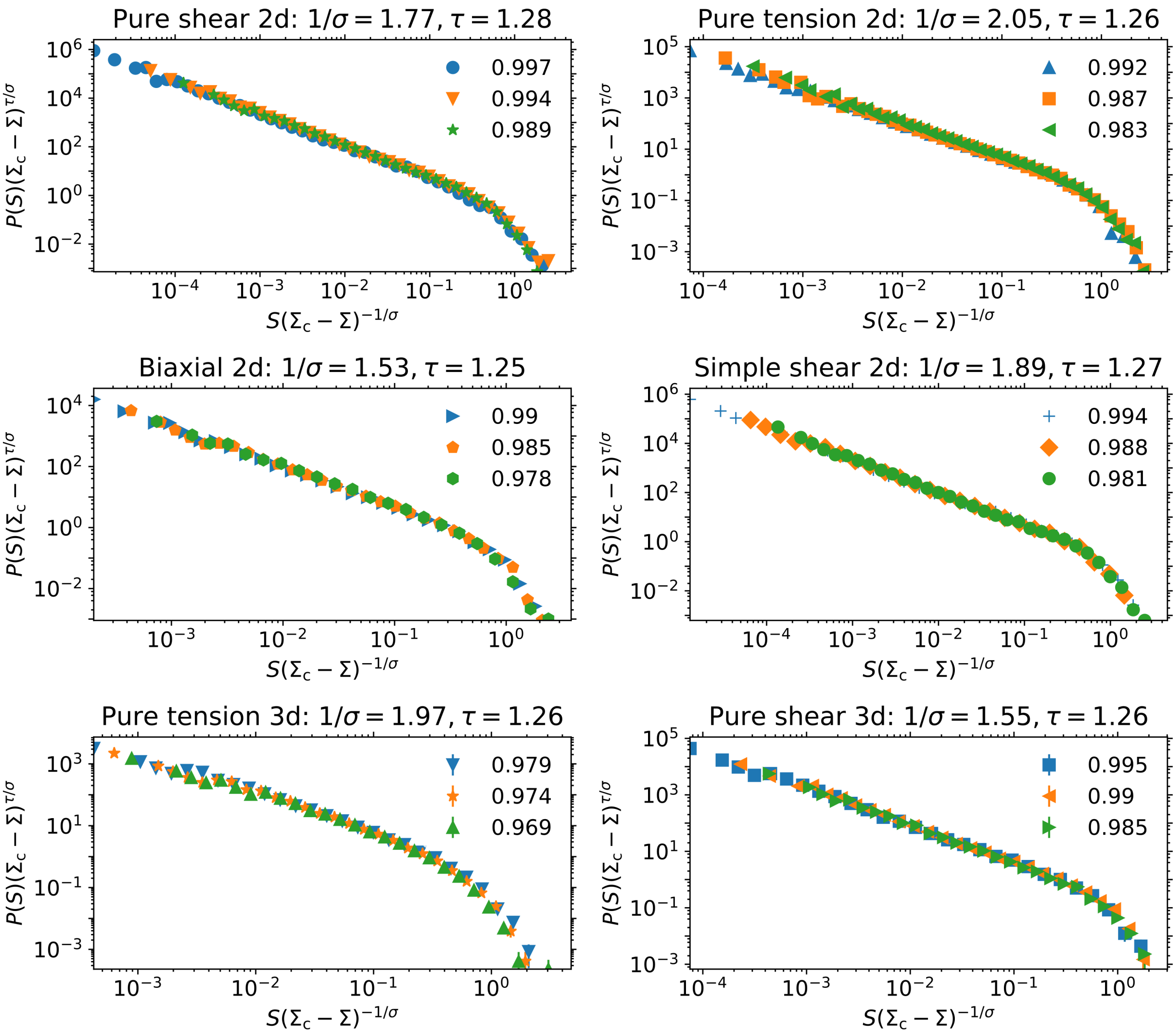

Supplementary Figure 1: **Data collapse for avalanche size distributions.** The distributions have been fitted jointly for different applied stresses but separately for each loading condition using Eqs. (2,3) of the main text. Values in the legends are applied stresses as a fraction of the critical stress Σ_c. Coupling constant $\mathcal{C} = 0.05$ for all simulations.

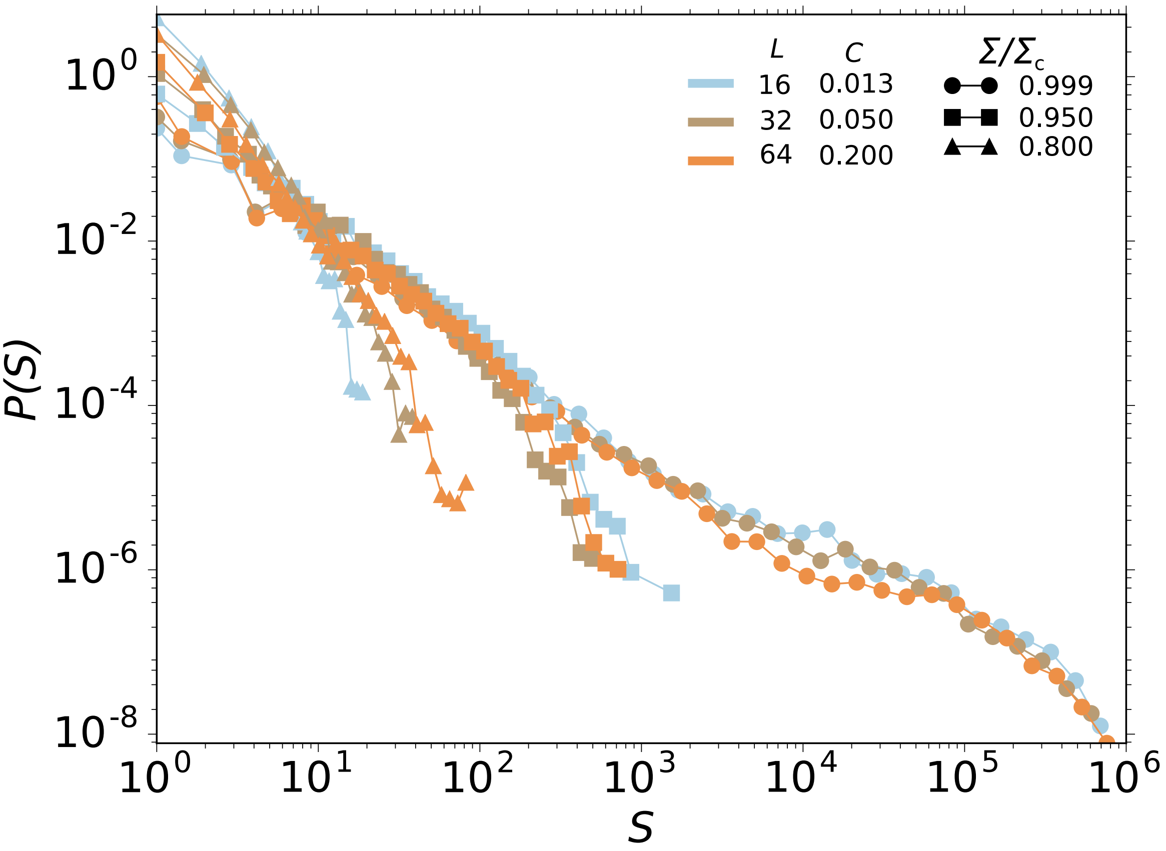

Supplementary Figure 2: **Avalanche distributions are invariant under change of model resolution.** Linear size L and coupling constant $\mathcal{C}$ are chosen to maintain $L^D/\mathcal{C} = \text{const}$. Simulations are performed in simple shear in $D = 2$, and distributions are given for three different values of the reduced stress.

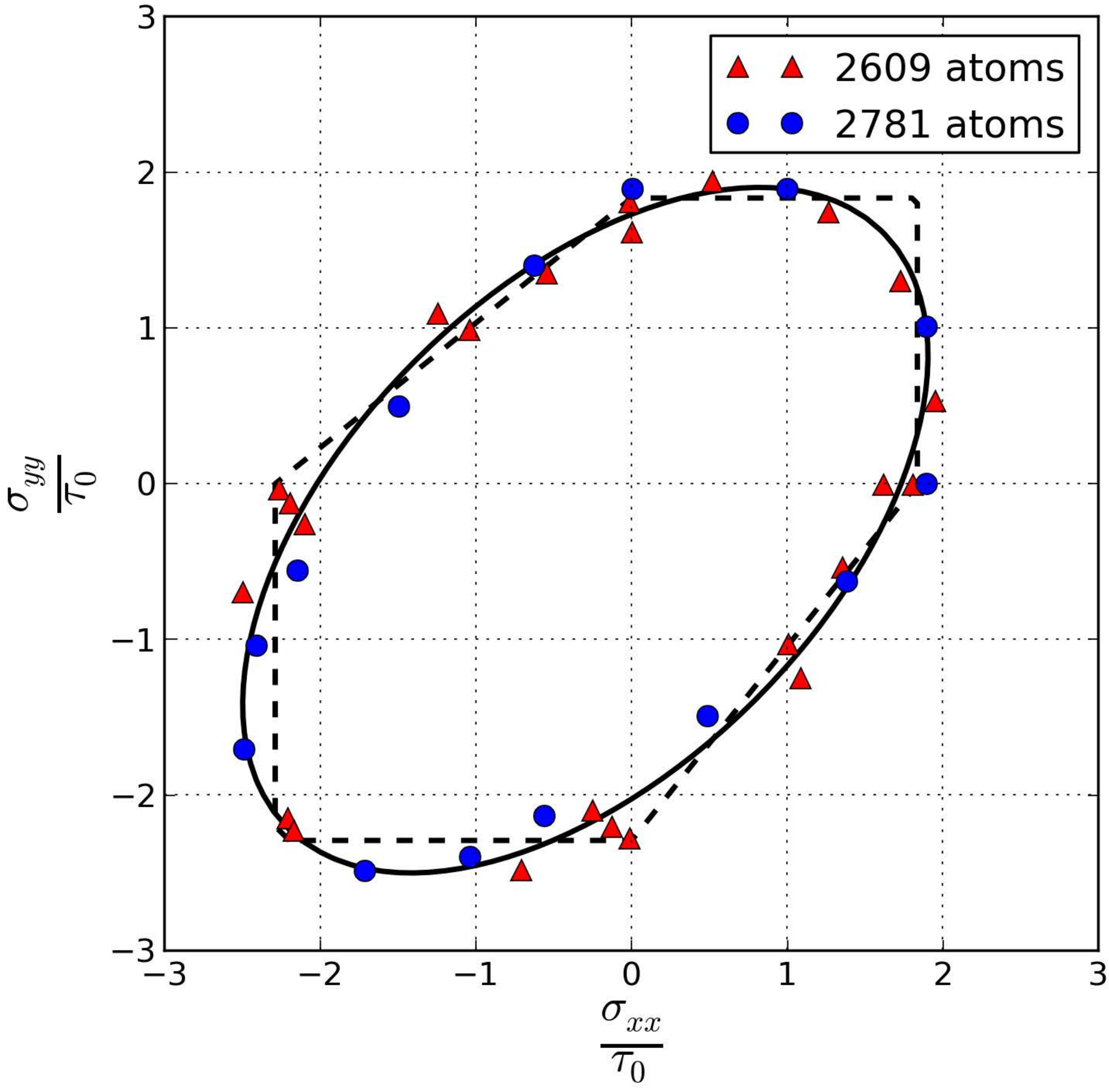

Supplementary Figure 3: **Yield surface of a metallic glass.** The yield surface is deduced from MD simulations of biaxial loading. Fits are a Mohr-Coulomb yield criterion (dashed line) and a Drucker-Prager yield criterion given in Eq. (7) of the main text with $\alpha = 0.085$ (solid line). The yield surface data and Mohr-Coulomb fit are taken from Schuh and Lund (Atomistic basis for the plastic yield criterion of metallic glass. *Nature Materials*, 2:449–452, 2003).

Supplementary Table 1: **Nonuniversal fitted yield stress.** The values of Σ_c are obtained from fits using Eqs (2,3) of the main text for the loading conditions shown in Fig. 2 of the main text.

Loading	Σ_c
Pure shear, 2D	$0.57746 \pm 7 \times 10^{-5}$
Pure tension, 2D	$1.13500 \pm 9 \times 10^{-5}$
Biaxial, 2D	$0.4124 \pm 3 \times 10^{-4}$
Simple shear, 2D	$0.31591 \pm 8 \times 10^{-5}$
Pure tension, 3D, PBCs	$0.9390 \pm 3 \times 10^{-4}$
Pure shear, 3D, PBCs	$0.4944 \pm 2 \times 10^{-4}$

Supplementary Table 2: **Fitted values of γ and a.** The values of the exponent γ and asymmetry parameter obtained by fitting the curves shown in Fig. 5 of the main text, using the form of Eq. (5) of the main text. Where a values have been left blank, the asymmetry is too small to be measured by fitting the curves.

Loading	γ	a
Pure tension, 3D	1.74 ± 0.02	–
Pure shear, 3D	1.74 ± 0.02	–
Simple shear, 2D	1.86 ± 0.02	–
Bending, 2D	1.77 ± 0.02	0.11 ± 0.03
Indentation, 2D	1.87 ± 0.02	0.15 ± 0.03
